# Natural flavonoids as potential multifunctional agents in prevention of diabetic cataract

**DOI:** 10.2478/v10102-011-0013-y

**Published:** 2011-06

**Authors:** Milan Stefek

**Affiliations:** Institute of Experimental Pharmacology & Toxicology, Slovak Academy of Sciences, SK-84104 Bratislava, Slovak Republic

**Keywords:** diabetic cataract, natural flavonoids, oxidative stress, antioxidans, aldose reductase inhibitors, glycation inhibitors

## Abstract

Cataract is one of the earliest secondary complications of diabetes mellitus. The lens is a closed system with limited capability to repair or regenerate itself. Current evidence supports the view that cataractogenesis is a multifactorial process. Mechanisms related to glucose toxicity, namely oxidative stress, processes of non-enzymatic glycation and enhanced polyol pathway significantly contribute to the development of eye lens opacity under conditions of diabetes. There is an urgent need for inexpensive, non-surgical approaches to the treatment of cataract. Recently, considerable attention has been devoted to the search for phytochemical therapeutics. Several pharmacological actions of natural flavonoids may operate in the prevention of cataract since flavonoids are capable of affecting multiple mechanisms or etiological factors responsible for the development of diabetic cataract. In the present paper, natural flavonoids are reviewed as potential agents that could reduce the risk of cataract formation via affecting multiple pathways pertinent to eye lens opacification. In addition, the bioavailability of flavonoids for the lens is considered.

## Introduction

Diabetic patients are susceptible to the development of chronic health complications responsible for a significant increase in their morbidity and mortality. Cataract, eye lens opacification, is one of the earliest secondary complications of diabetes mellitus (Kyselova *et al*., 2004; Obrosova *et al*., 2010). Diabetic patients are about 60% more likely to develop these eye conditions. People with diabetes also tend to get cataract at a younger age with a faster progression (Bron *et al*., 1998).

The association between diabetes and cataract formation has been shown in clinical epidemiological and basic research studies (Bron *et al*., 1998; Pollreisz and Schmidt-Erfurth, 2010; Obrosova *et al*., 2010). Since extracellular glucose diffuses into the lens uncontrolled by the hormone insulin, the lens is one of the most affected body parts in diabetes mellitus. The understanding of mechanisms by which glucose exerts its toxicity is of utmost importance for rational pharmacological interventions to treat diabetic cataract. The etiology of diabetic cataract is multifactorial; multiple hyperglycemia-dependent mechanisms leading to an array of subtle post-translational modifications in the lens structural proteins contribute to its development. The lens is a closed system with limited capability to repair or regenerate itself. The proteins of the lens are extremely long-lived and there is virtually no protein turnover, which provides great opportunities for post-translational modification to occur (Bron *et al*., 2000; Harding, 2002; Krishna Sharma & Santhoshkumar, 2009). Oxidative stress and its sequelae are clearly involved in the etiology of senile cataract, while mechanisms related to glucose toxicity, namely oxidative stress, enhanced polyol pathway, and processes of non-enzymatic glycation, significantly contribute to the development of eye complications in diabetic patients (Baynes & Thorpe, 1999; Kyselova *et al*., 2004; Brownlee, 2005; DelCorso *et al*., 2008; Alexiou *et al*., 2009; Obrosova *et al*., 2010).

Under conditions of diabetes, the need of tight blood glucose control is a key prerequisite to reduce the incidence, progression, and severity of cataract. Yet periods of hyperglycemia in the daily regimen of a diabetic patient cannot be avoided, with all of the aforementioned deleterious consequences of glucose toxicity. Therefore additional adjunct therapy interfering with the pathological processes at molecular level, *e.g.* based on antioxidants, aldose reductase inhibitors and anti-glycation agents, is needed to attenuate the noxious effects of glucose.

Innovative strategies in treatment of diseases of multifactorial origin are oriented on a rational design of chemical entities able to affect simultaneously multiple key mechanisms involved. This approach increases the chance of successful therapeutic intervention, decreases the risk of side effects and is economical. An example of the "multi-target" strategy in treatment of diabetic complications are the bifunctional compounds combining the aldose reductase inhibiory activity with the antioxidant effect, including pyrido-pyrimidines (La Motta *et al*., 2007), pyridazines (Coudert *et al*., 1994), benzopyranes (Constantino *et al*., 1999) and carboxymethylated pyridoindoles (Stefek *et al*., 2008) or compounds combining the aldose reductase inhibiory activity with the ability to attenuate nonenzymatic glycation (Demopoulos *et al*., 2005). Considering the detoxification role of aldose reductase against toxic carbonyl products, significantly enhanced in diabetic tissues (Baynes, 1991; Thorpe & Baynes, 1996; Thornalley, 2005; Turk, 2010), the concurrent antioxidant action of a multifunctional drug can counterbalance its inhibition. In addition, antioxidant activity may suppress processes of advanced glycation (glycoxidation) at the level of free radical intermediates (Baynes, 1991; Thorpe & Baynes, 1996; Brownlee, 2000; Giacco & Brownlee, 2010).

Recently, considerable attention has been devoted to the search for phytochemical therapeutics. There is epidemiologic evidence that a sufficient intake of fruit and vegetables can lower the risk of cataract in humans (Taylor, 1993, 1999). A variety of constituents, like vitamins, minerals, fiber, and numerous phytochemicals, including flavonoids may contribute to the protective effect of fruits and vegetables. Indeed, several pharmacological actions of flavonoids may operate in the prevention of both age-related and diabetic cataract since flavonoids are capable of affecting multiple mechanisms or etiological factors responsible for the development of sight threatening ocular diseases (Bhimanagouda, 2009; Majumdar *et al*., 2010; Kalt *et al*. 2010)

**Figure 1:**
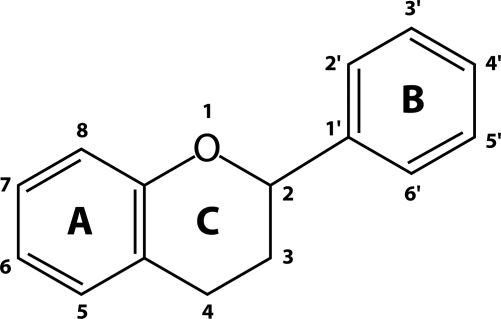
Basic flavonoid structure.

At the pre-clinical level of animal models, flavonoids (Figure 1) have been shown to be protective against eye lens opacification. In the present paper, flavonoids are reviewed as potential agents that could reduce the risk of cataract formation via affecting multiple key pathways pertinent to eye lens opacification, including oxidative stress, non-enzymatic glycation and polyol pathway. In addition, the bioavailability of flavonoids to the lens is considered.

## Anticataract action of flavonoids

### Models *in vitro*

Under *in vitro* conditions, using rat lens organ culture endowed with hydrogen peroxide, low micromolar levels of flavonol quercetin inhibited oxidation-induced sodium and calcium influx and loss of lens transparency (Sanderson *et al*., 1999). As shown later by Cornish *et al*. (2002), quercetin was rapidly lost from the media and readily entered the lens where it was methylated to 3′-O-methyl quercetin. Both quercetin and its metabolite were active in inhibiting oxidative damage in the lens.

The glucoside of isorhamnetin (methylated quercetin), isolated as a bioactive flavonoid from the leaves of *Cochlospermum religiosum* (Gayathri *et al*., 2010) and flavonoid fraction isolated from fresh leaves of *Vitex negundo* (Rooban *et al*., 2011) protected enucleated rat eye lenses against selenite-induced cataract in an *in vitro* culture model.

The flavonoid venoruton, a mixture of mono-, di-, tri- and tetrahydroxyethylrutosides, significantly reduced the degree of opacification and the leakage of lactate dehydrogenase in rat lens organ culture simulating diabetic conditions (Kilic *et al*., 1997).

### Animal models *in vivo*

As early as in 1977, Varma *et al*. (1977) studied the effect of quercetin rhamnoside (quercitrin) on the development of cataract in the rodent *Octodon degus* made diabetic by a single intraperitoneal dose of streptozotocin. The control diabetic animals not receiving quercitrin developed nuclear opacity by about the tenth day after the onset of hyperglycemia. In contrast, the diabetic animals treated with quercitrin did not develop cataracts even 25 days after the onset of diabetes, although they had a blood glucose concentration similar to that of the control diabetic group. In a similar study performed by Lu *et al*. (2008) in streptozotocin diabetic rats, high-isoflavone soy protein markedly decreased the death rate and incidence of cataracts in the diabetic animals. At the same time, reduced serum glucose and methylglyoxal were recorded in the treated rats. Nakano *et al*. (2008) reported lower incidence of cataract in streptozotocin diabetic rats treated with flavangenol, acomplex mixture of bioflavaonoids with oligomeric proanthocyanidin as main constituents.

Topical administration of quercetin to the orbital pouch of the galactosemic neonatal rat diminished cataractogenesis in the corresponding lens. Comparison with the contralateral lens indicated that quercetin reduced intracellular edema, prevented extracellular fluid accumulation and maintained cellular interdigitation of the superficial anterior cortical fiber. In addition to preserving fiber integrity, topical application of quercetin maintained lens growth as evidenced by radius and dry weight measurements (Beyer-Meyers & Farnsworth, 1979). Analogically, in the same galactosemic rat model, Mohan *et al*. (1988) recorded anticataract action of quercetin and structurally related myricetin after topical administration. In galactosemic rats, oral treatment with quercetin resulted in a significant correction of eye lens electrolyte disturbances and normalization of lens protein levels (Ramana *et al*., 2007). The results imply that inclusion of quercetin contributes to lens transparency through the maintenance of characteristic osmotic ion equilibrium and protein levels of the lens. The isoflavone genistein delayed the progression of cataracts induced in rats by dietary galactose (Huang *et al*., 2007).

The rat selenite cataract model (Shearer *et al*., 1997; Ghupta *et al*., 2009; Kyselova, 2010) was extensively used to study the anticataract action of flavonoids. The results of Orhan *et al*. (1999) showed that ethanol extract of propolis, rich in flavonoids (Scheller *et al*., 1990), and quercetin prevented cataract formation to the extent of 70 and 40%, respectively. Standardized extract of *Ginkgo biloba* (Egb761) did not affect cataract formation. The flavonoid fraction from *Emilia sonchifolia* was reported (Lija *et al*., 2006) to decrease the rate of maturation of selenite cataract more efficiently than quercetin. Activities of superoxide dismutase, catalase and reduced glutathione were found to be increased in the group treated with *Emilia sonchifolia*, while thiobarbituric acid reacting substances were decreased compared with the selenite induced group.

Rutin (quercetin rutinoside) was reported (Isai *et al*., 2009) to prevent selenite-induced cataractogenesis in rat pups. At the end of a 30-day study period, all the rat pups that had received only selenite were found to have developed a dense nuclear opacity in the lens of each eye, whereas only 33.3% of pups that had received selenite and been treated with an intraperitoneal dose (175 mg/kg of body weight) of rutin hydrate were found to have mild lenticular opacification in each eye. The other 66.7% of pups in that group had clear lenses in both eyes, as in normal pups. The mean activities of catalase, superoxide dismutase, glutathione peroxidase, glutathione transferase, and glutathione reductase were found to be significantly lower in the lenses of cataract-untreated rat pups than in normal control rat lenses. However, in lenses treated with rutin hydrate, the mean activities of antioxidant enzymes were significantly higher than the values in rat pups with untreated cataracts.

Onion is a flavonoid-rich foodstuff and the major flavonoids contained have been identified as quercetin, quercetin-4‘-glucoside and quercetin-3,4‘-diglucoside (Fossen *et al*., 1998; Miean and Mohamed, 2001). In the study of Javadzadeh *et al*. (2009), the instillation of fresh juice of crude onion into the rat eyes was found to prevent selenite-induced cataract formation by 75%. This effect was associated with higher mean total antioxidant level as well as higher mean activities of superoxide dismutase and glutathione peroxidase in the lenses of rats receiving fresh juice of crude onion and subcutaneous injection of sodium-selenite, compared with those rats which received only sodium-selenite injection. The onion juice, as a flavonoid-rich source, was postulated to provide an additional support to the antioxidant agents, leading to the elevation of total antioxidant levels and superoxide dismutase and glutathione peroxidase activities in the rat lens, in spite of exposure to sodium-selenite.

Flavonoid fractions isolated from natural sources including green and black tea (Thiagarajan *et al*., 2001; Gupta *et al*., 2002), *Ginkgo biloba* (Thiagarajan, 2002), grape seeds (Durukan *et al*., 2006), *Emilia sonchifolia* (Lija *et al*., 2006), *Vitex negundo* (Rooban *et al*. 2009) and broccoli (Vibin *et al*., 2010) were shown to have anticataract activity in selenite-induced experimental cataract in rats. In addition, *Ginkgo biloba* extracts were found to protect rats against radiation-induced cataract (Ertekin *et al*., 2004).

Among others, damage to the lens epithelium is considered a possible cause of cataract formation (Hightower, 1995). Catechin was found to inhibit apoptotic cell death in the lens epithelium of rats after cataract induction with N-methyl-N-nitrosourea (Leed *et al*., 2010). Grape seed extract rich in flavonoids reduced hydrogen-peroxide-induced apoptosis of human lens epithelial cells and depressed H_2_O_2_-induced activation and translocation of NF-kB (Jia *et al*., 2011). Similarly, the flavonoid fisetin was found to protect human lens epithelial cells from UVB-induced oxidative stress by inhibiting the generation of reactive oxygen species and modulating the activation of NF-kB and MAPK pathways (Yao *et al*., 2008).

## Flavonoids as multifunctional agents

As reviewed below, flavonoids efficiently affect the multiple key molecular mechanisms involved in the etiology of both age-related and diabetic cataract, namely oxidative stress, non-enzymatic glycation and polyol pathway. Structural requirements of flavonoids for efficient inhibition of the above mentioned processes are summarized. Flavonoids may interfere also with lens calpain proteases and lens epithelial cell signaling, which is however outside the scope of this review.

### Antioxidant action of flavonoids

The antioxidant action of flavonoids, the best described biological activity of this group of natural polyphenolic substances, is covered by a number of excellent reviews (Bors *et al*., 1990; Cao *et al*., 1997; Pietta, 2000; Rice-Evans, 2001; Nijveldt *et al*., 2001; Bors & Michel, 2002; Heim *et al*., 2002; Williams *et al*., 2004; Amić *et al*., 2007; Bischoff, 2008; Boots *et al*., 2008). Flavonoids may exert antioxidant effects due to their ability to act as free radical scavengers, hydrogen donating compounds, singlet oxygen quenchers, and metal ion chelators. Within the flavonoid family, quercetin is the most potent scavenger of reactive oxygen species, including superoxide, peroxyl, alkoxyl and hydroxyl radicals, and reactive nitrogen species like NO^.^ and ONOO (Pietta, 2000; Butković *et al*., 2004; Amić *et al*., 2007; Boots *et al*., 2008). It is beyond the scope of this review to give a thorough survey of the abundant literature covering numerous studies of the antioxidant action of flavonoids. Nevertheless, key structural features responsible for the high antioxidant efficacy of flavonoids, also with relevance to the eye lens, are highlighted.

The general structural requirements for effective radical scavenging and/or the antioxidant potential of flavonoids are known as Bors’ criteria (Bors & Michel, 2002; Amić *et al*., 2007), namely (i) the presence of a catechol group in ring B, capable of readily donating hydrogen (electron) to stabilize a radical species, (ii) the presence of 2,3-unsaturation in conjugation with a 4-oxo-function in the C-ring, which is responsible for electron delocalization, and (iii) the presence of a 3-hydroxyl group in the heterocyclic ring which increases the radical-scavenging activity. The catechol moiety may also contribute to an ability to chelate transition metal ions, such as copper and iron.

Flavonoids were found also to scavenge efficiently the model free radicals of 2,2-diphenyl-1-picrylhydrazyl and α,γ-bisdiphenylene-β-phenylallyl (ButkoviČ *et al*., 2004). Flavonoid chelating activity for transition metal ions has been well documented (Nijveldt *et al*. 2001; Pietta, 2000;Williams *et al*., 2004). Flavonoids inhibit xanthine oxidase, the enzyme responsible for superoxide anion production (Hamasaki *et al*., 1994). Interestingly, isorhamnetin (3-methylquercetin) was found to inhibit xanthine oxidase, even more efficiently than the aglycone form of quercetin (Nagao *et al*., 1999).

As reviewed below, the high antioxidant efficacy of flavonoids is accompanied by their ability to inhibit aldose reductase and non-enzymatic glycation – activities of high relevance to the development of diabetic cataract.

### Aldose reductase inhibition by flavonoids

The accumulation of polyol sorbitol within the lens is a primary contributing factor to the formation of diabetic cataract (Yabe-Nishimura, 1998; Del Corso *et al*., 2008; Alexiou *et al*., 2009; Obrosova, 2010), a mechanism different from senile cataract. In diabetes, glucose is in a high concentration in the aqueous humor and can diffuse passively into the lens. The enzyme aldose reductase within the lens converts glucose to sorbitol. This polyol cannot diffuse passively out of the lens and accumulates or is converted to fructose.

Aldose reductase inhibitors represent a potential therapeutic strategy for preventing the onset or progression of diabetic cataract (Costantino *et al*., 2000; Miyamoto, 2002; Suzen *et al*. 2003; Alexiou *et al*., 2009; Obrosova, 2010). Pharmacophoric requirements for aldose reductase inhibitors are determined by the structural features of the inhibitor binding site of aldose reductase, which was shown to be formed by a large hydrophobic pocket (El-Kabbani *et al*., 2004). This pocket is mainly composed of two regions: a hydrophilic anionic binding site which accommodates acidic functionalities and a region of hydrophobic residues that binds the hydrophobic aromatic ring system of the inhibitors. Inhibitor binding is therefore a consequence of polar and non-polar interactions between the inhibitor and the complementary residues that line the enzyme binding pocket. It has been proposed that the specificity for the inhibitor was mainly due to inhibitor-enzyme interactions at the non-polar domain (El-Kabbani & Podjarny, 2007).

To date, two main classes of active aldose reductase inhibitors have been reported and classified on the basis of the ionizable group which allows them to anchor to the catalytic site: carboxylic acids (substitution derivatives of acetic acid) and spirohydantoins, with epalrestat and sorbinil being the most representative members of each respective family (Costantino *et al*., 2000; Miyamoto, 2002; Suzen *et al*. 2003; Alexiou *et al*., 2009). They are generally referred to as carboxylate-type and hydantoin-type inhibitors. A third class of aldose reductase inhibitors represents flavonoids. Since the mid-1970s, a number of studies have been reported on the inhibition of aldose reductase by flavonoids (Varma *et al*., 1975; Varma and Kinoshita, 1976; Okuda *et al*., 1982; Nakai *et al*., 1985; Lim *et al*., 2001; Jung *et al*., 2002; Matsuda *et al*., 2002; Kawanishi *et al*., 2003; Lee *et al*., 2010). Structural features required for a firm anchoring to the catalytic site of the aldose reductase enzyme, were summarized by Matsuda *et al*. (2002) as follows: (i) the presence of a 7-hydroxyl group and catechol moiety at the B ring guarantees the strong activity; (ii) the 5-hydroxyl moiety does not affect the activity; (iii) the 3-hydroxyl and 7-O-glucosyl moieties reduce the activity; (iv) the 2–3 double bond enhances the activity; (v) the flavonols having the catechol moiety (the 3′,4′-dihydroxyl moiety) at the B ring exhibit stronger activity than those with the pyrogallol moiety (the 3′,4′,5′-trihydroxyl moiety).

Inhibitory action of active flavonoid components isolated from natural products against rat lens or human recombinant aldose reductase were reported, often in comparison with quercetin used as a positive control (Matsuda *et al*., 2002; Suryanarayana *et al*., 2004; Wirasathien *et al*., 2007; Chethan *et al*., 2008; Carbone *et al*., 2009; Jung *et al*., 2008a, b 2009, Jung *et al*., 2011; Lee *et al*., 2010).

Aldose reductase inhibitors of the flavonoid class, in contrast to those of the carboxylate type whose acidic nature results in poor biological availability, possess a higher pK_a_ value, which is a prerequisite for their better pharmacokinetics.

In the light of the above mentioned biological activities of natural flavonoids, they serve as an example of bifunctional agents for the "multi-target" approach to the treatment of diabetic cataract by combining the aldose reductase inhibitory activity with its antioxidant action. In addition, starting from the flavonol quercetin as a lead structure, a series of 4H-1-benzopyran-4-one derivatives was designed and developed as semi-synthetic agents, with dual antioxidant/ aldose reductase inhibition activity (Costantino *et al*., 1999).

### Advanced glycation inhibition by flavonoids

The process of non-enzymatic glycation is well known to be one of the key mechanisms leading to diabetic cataract (Shamsi *et al*., 2000; Brownlee, 2001, 2005; Stitt, 2005; Monnier *et al*. 2005; Nagaraj *et al*., 2010). In compliance with the glycation theory of aging (Monnier & Cerami, 1981), accumulation of advanced glycation endproducts in the aging lens, yet to a lesser extent in comparison with the diabetic eye, may contribute to age-related lens opacity. In seeking potential anti-cataract drugs, clinically useful anti-glycation agents are a reasonable option. As reviewed below, a number of naturally occurring flavonoids were reported to exhibit inhibitory effects on advanced glycation endproducts formation.

Four flavonoids of the methanol extract of *Thymus vulgaris*, quercetin, eriodictyol, 5,6,4’-trihydroxy-7,8,3’-trimethoxyflavone, and cirsilineol suppressed the levels of advanced glycation end products and fructosamines of bovine serum albumin under *in vitro* conditions (Morimitsu *et al*., 1995). By using the bovine serum albumin glycation model, Beaulieu *et al*. (2010) demonstrated that the flavonoid components of the *Vaccinium vitis-idaea* berry extract were potent antiglycation agents, while Wu *et al*.(2005) recorded significant inhibitory activity of the natural flavonoids luteolin, quercetin, and rutin. Rutin and metabolites of rutin were found to inhibit glycation product formation, including both fluorescent and nonfluorescent AGEs, induced by glucose in collagen I *in vitro* (Cervantes-Laurean, 2006). Flavonoids markedly reduced pentosidine formation in collagen from bovine Achilles tendon incubated with glucose *in vitro* in the following decreasing order of their specific inhibitory activity: myricetin ≥ quercetin> rutin>(+)catechin>kaempferol (Urios *et al*., 2007). Rutin and its metabolites 3,4-dihydroxyphenylacetic acid and 3,4-dihydroxytoluene were found to inhibit histone H1 glycation by the powerful glycating agent ADP-ribose, as effectively as did aminoguanidine (Pashikanti *et al*., 2010). Rutin and G-rutin, a water soluble glucose derivative of rutin, suppressed glycation of muscle and kidney proteins exposed to glucose *in vitro* (Nagasawa *et al*., 2003a). Under *in vivo* conditions, G-rutin was found to inhibit glycation reactions in muscle, kidney and plasma proteins of streptozotocin-induced diabetic rats (Nagasawa *et al*., 2003b).

In their study based on 62 flavonoids, Matsuda *et al*. (2003) formulated structural requirements of flavonoids for inhibition of protein non-enzymatic glycation: (i) as the hydroxyl groups at the 3′-, 4′-, 5-, and 7-positions increased in number, the inhibitory activities became stronger; (ii) the activities of flavones were stronger than those of corresponding flavonols, flavanones, and isoflavones; (iii) methylation or glucosylation of the 4′-hydroxyl group of flavones, flavonols, and flavanones reduced their activity; (iv) methylation or glycosylation of the 3-hydroxyl group of flavonols tended to increase activity; (v) glycosylation of the 7-hydroxyl group of flavones and isoflavones reduced their activity. Yet these principles should be further corroborated.

In seeking more efficient multifunctional flavonoids combining antioxidant activity with both aldose reductase and advanced glycation inhibitory action, for potential pharmacological prevention of diabetic cataract and other long-term diabetic complications, all three sets of the aforementioned criteria should be applied in screening available databases of flavonoid structures.

## Bioavailability of flavonoids

Biological activity of flavonoids is often assessed by using *in vitro* models; in almost all such studies, cells are treated with aglycones and data are reported at concentrations that elicited a response. However, plasma and tissues are not exposed *in vivo* to flavonoids in these forms. The forms reaching the blood and tissues are, in general, neither aglycones nor the same as the dietary source glycosides. In blood, flavonoids are present as conjugates of glucuronate or sulfate, with or without methylation of the catechol functional group. As a consequence, the flavonoid conjugates are likely to possess different biological properties and distribution patterns within tissues and cells than have flavonoid aglycones. Although deconjugation can potentially occur *in vivo* to produce aglycone, it occurs only at certain sites. Thus, the extent to which *in vitro* effects produced by the aglycones can be extrapolated to the *in vivo* situation, in particular in humans, is poorly understood (Kroon *et al*., 2004).

Flavonoids occur in plants mainly in the form of O-glycoside conjugates linked to sugars like glucose, galactose, arabinose or rhamnose (Bravo, 1998; Arts *et al*., 2004). The bioavailability is primarily determined by the type of the sugar moiety (Arts *et al*., 2004; Crozier *et al*., 2010). In the case of flavonoid-O-β-D-glucosides, the aglycone can be enzymatically released in the small intestine by the brush border lactase phlorizin hydrolase (Day *et al*., 1998) or by cytosolic β-glucosidase activity (Ioku *et al*., 1998). The enzyme of lactase phlorizin hydrolase exhibits broad substrate specificity for flavonoid-monoglucosides and the released aglycone may then enter the small intestine epithelial cells by passive diffusion (Day *et al*., 2000). Alternatively, cytosolic β-glucosidase functions within the epithelial cells after the glucosides entered via the sodium-dependent glucose transporter 1(Gee *et al*., 2000). On the other hand,*e.g.* quercetin-3-rutinoside (rutin) is not a substrate of lactase phlorizin hydrolase. The quercetin aglycone is released hydrolytically by bacterial α-rhamnosidases and β-glucosidases in the lower gastrointestinal tract. Thus absorption of quercetin from rutin is delayed, and quercetin bioavailability is much smaller in comparison with that of quercetin-glucosides. This example stresses the role of sugar type for bioavalability of natural flavonoid glycosides (Hollman *et al*., 1999; Jaganath *et al*., 2006; Manach *et al*., 2005; Crozier *et al*., 2010). Prior to passage into the blood stream, the aglycones undergo metabolism forming sulfate, glucuronide and/or methylated metabolites (Morand *et al*., 1998; Manach *et al*., 2005; Mullen *et al*., 2006; Crozier *et al*., 2010; Kay, 2010).

Excellent reviews covering broadly the topic of bioavalability of flavonoids were published (Scalbert & Williamson, 2000; Scalbert *et al*., 2002; Wale, 2004; Clifford, 2004; Manach *et al*., 2005; Wiliamson and Manach, 2005; Crozier *et al*., 2009, Crozier *et al*., 2010; Kay, 2010).

To date, only a few studies have investigated delivery of flavonoids to the eye. Drug delivery to the ocular tissues depends on the physicochemical and biopharmaceutical characteristics of the selected flavonoids and very importantly on the route of administration. Topical application is the most favored mode for ocular conditions. The topical route is mainly used to deliver drugs to the anterior segment of the eye. Local administration may yield much higher and effective concentrations of the parent flavonoids in the ocular tissues and at much lower doses, than the oral route. In the anterior chamber of the eye, the aqueous humor bathes the anterior surface of the lens, providing all oxygen and nutrient requirements. It is therefore this route through which dietary flavonoids would reach the lens. For example, in the *ex vivo* experiments reported by Cornish *et al*. (2002), quercetin was shown to enter the lens. In the lens, enzymes which metabolized quercetin to 3′-O-methyl quercetin were identified. Metabolism reduced the efficacy but did not terminate the protective action of quercetin since 3′-O-methyl quercetin was also found to be effective in reducing opacification. In analogy with the ability of fluoroscein glucuronides to enter the anterior chamber of the eye following oral administration (Grotte *et al*., 1985), it was hypothesized that quercetin glucuronides would also be transported into the aqueous humor if present in plasma, where deglucuronidation could occur via endogenous β-glucuronidase activity. β-Glucuronidase is present in many tissues and body fluids in humans (Sperker *et al*., 1997) and its activity has been demonstrated in the normal human lens (Kamei, 1998) and aqueous humor (Weinreb *et al*., 1991). Following uptake of glucuronides into the aqueous humor, the lens could therefore be exposed to the circulating conjugates and/or to aglycone following deglucuronidation in the aqueous humor.

## Conclusions

Presently, surgical extraction remains the only cataract cure. Cataract surgery has become the most frequent surgical procedure in people aged 65 years or older in the Western world, causing a considerable financial burden to the health care system (Head, 2001; Meyer & Sekundo, 2005; Bockelbrink *et al*., 2008). Hence, there is an urgent need for inexpensive, non-surgical approaches to the treatment of cataract (Olson *et al*., 2003) since a delay of 10 years in the onset of cataract by any means would be expected to halve the number of cataract extractions (Brian & Taylor, 2001). This calls for a search of alternative pharmacological measures to treat this disorder.

In diabetic patients, tight control of hyperglycemia is the first prerequisite to attenuate the risk of cataract. In addition, adjunct therapy is needed to help preserve vision in diabetic patients, aimed at correcting biochemical and metabolic abnormalities in the hyperglycemic milieu of the diabetic individual.

The current data on natural polyphenols in relation to cataract, along with epidemiological knowledge on diet and lens opacity, demonstrate that flavonoids may play a role in cataract prevention. Yet future clinical trials are needed to assess the benefits of flavonoids in lowering the risk of both age-related and diabetic eye complications.
